# Supplementary material for: Synthesis, In Vitro Biological Evaluation of Antiproliferative and Neuroprotective Effects and In Silico Studies of Novel 16E-Arylidene-5α,6α-epoxyepiandrosterone Derivatives
Source: Biomedicines. 2023 Mar 7;11(3):812. doi: 10.3390/biomedicines11030812 (PMC10045663; doi:10.3390/biomedicines11030812)

Supplementary Material

Synthesis, In Vitro Biological Evaluation of Antiproliferative and Neuroprotective Effects and In Silico Studies of Novel 16E-Arylidene-5α,6α-epoxyepiandrosterone Derivatives

Vanessa Brito [1], Mariana Marques [1], Marta Esteves [1], Catarina Serra-Almeida [1], Gilberto Alves [1], Paulo Almeida [1], Liliana Bernardino [1], Samuel Silvestre [1,2*]

[1] Health Sciences Research Centre (CICS-UBI), Universidade da Beira Interior, Av. Infante D. Henrique 6200-506 Covilhã, Portugal

[2] Centre for Neurosciences and Cell Biology (CNC), University of Coimbra, 3004-517 Coimbra, Portugal

* Correspondence: sms@ubi.pt

Table of Contents	
NMR spectra of novel compounds	S1
Additional biological data	S2
Concentration-response curves for steroid **19** and IC_{50} determination additional data	S3
Interactome of steroid **22** at aromatase active site	S4

S1. NMR spectra of novel compounds

¹H NMR spectrum of compound 16

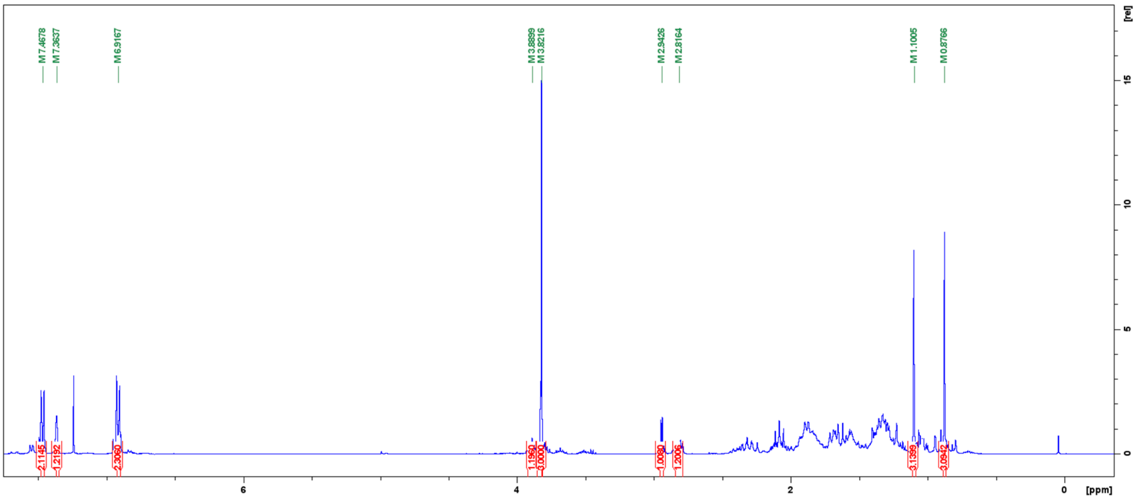

¹³C NMR spectrum of compound 16

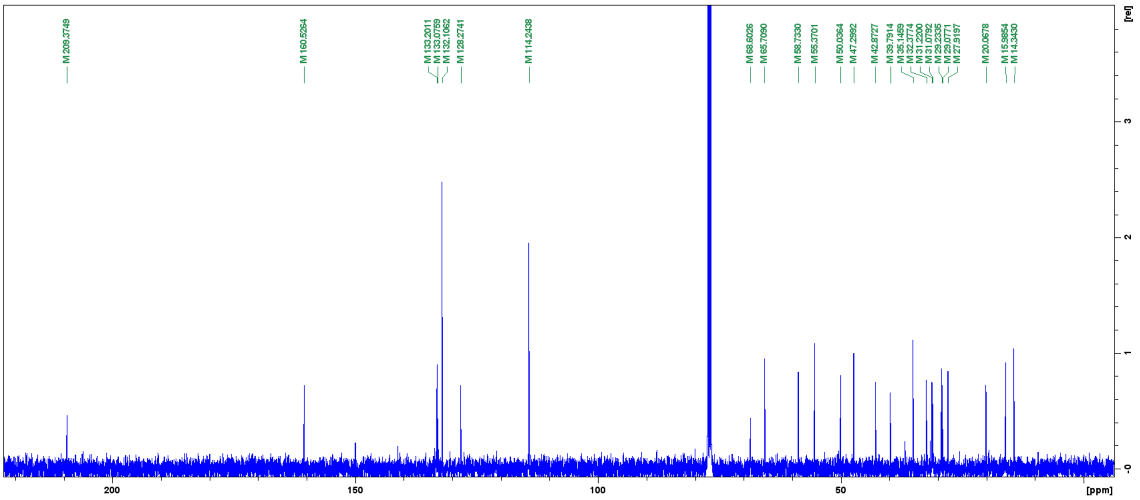

^{1}H NMR spectrum of compound 17

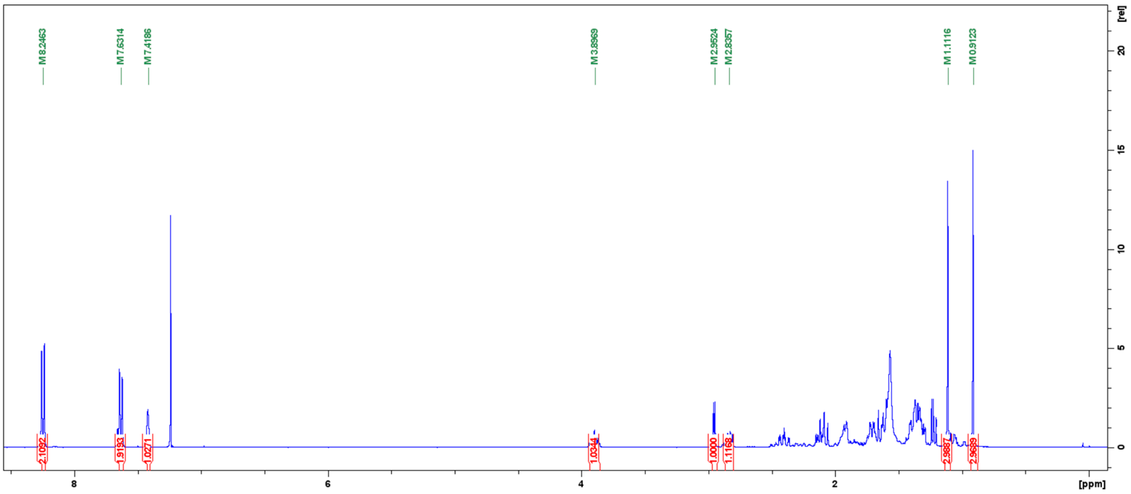

^{13}C NMR spectrum of compound 17

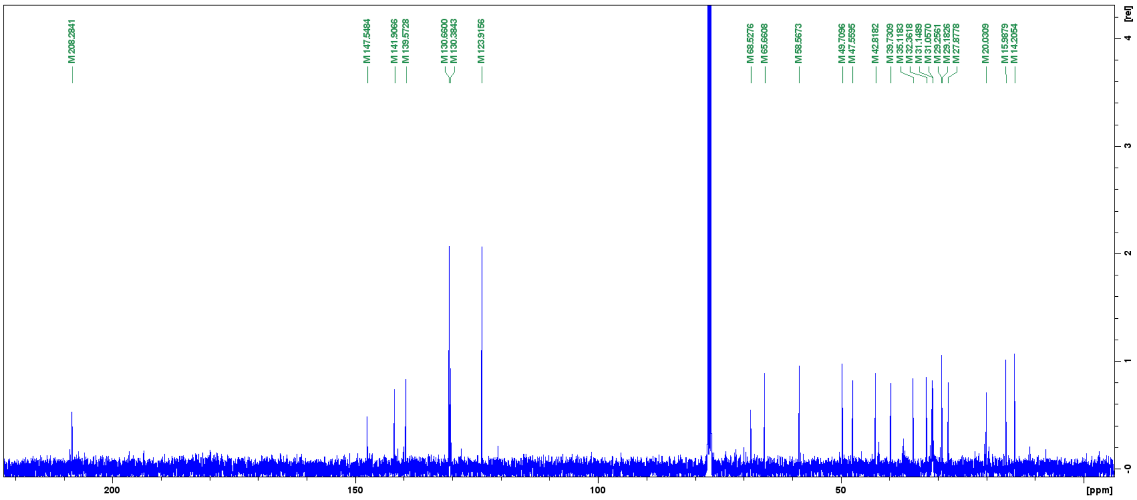

^{1}H NMR spectrum of compound 18

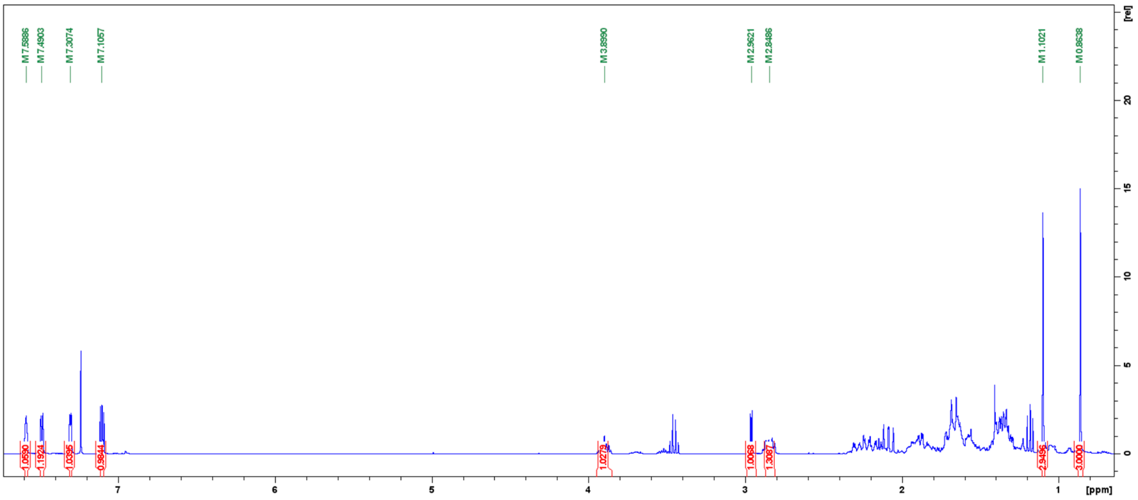

^{13}C NMR spectrum of compound 18

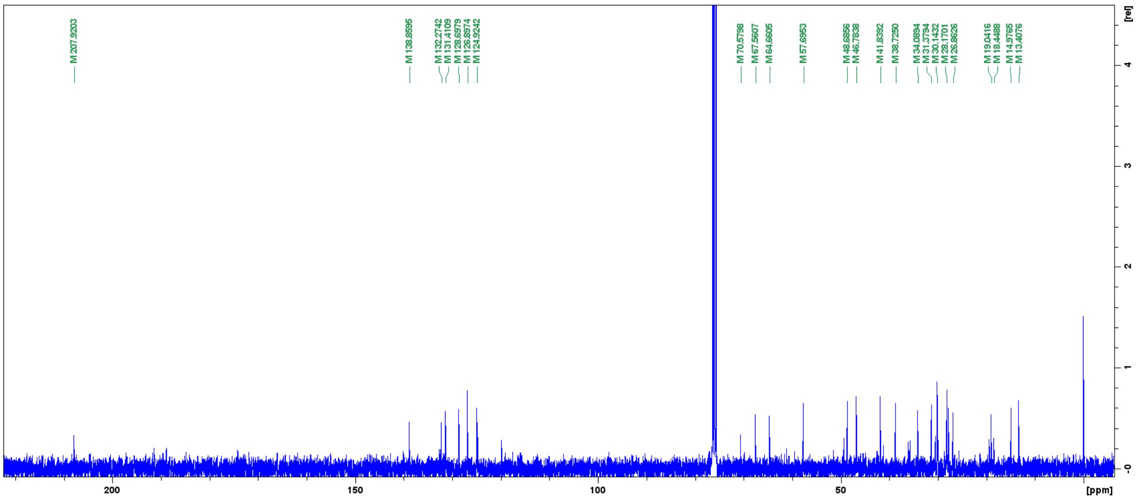

^{1}H NMR spectrum of compound 19

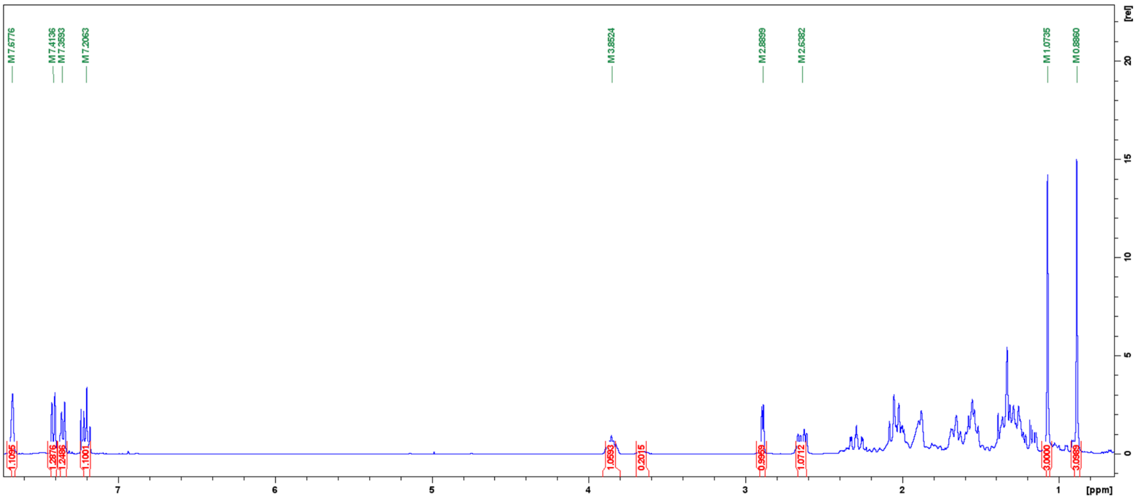

^{13}C NMR spectrum of compound 19

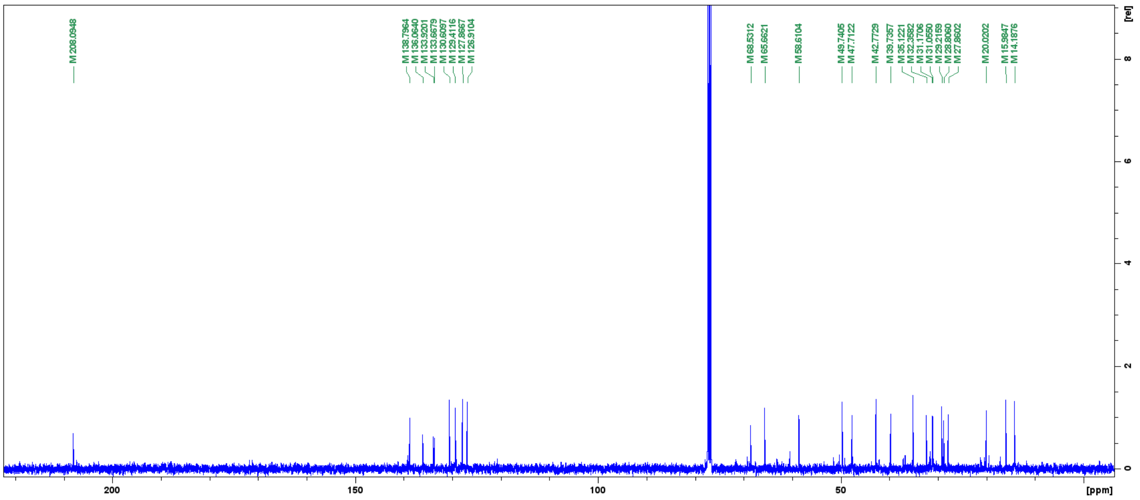

^{1}H NMR spectrum of compound 20

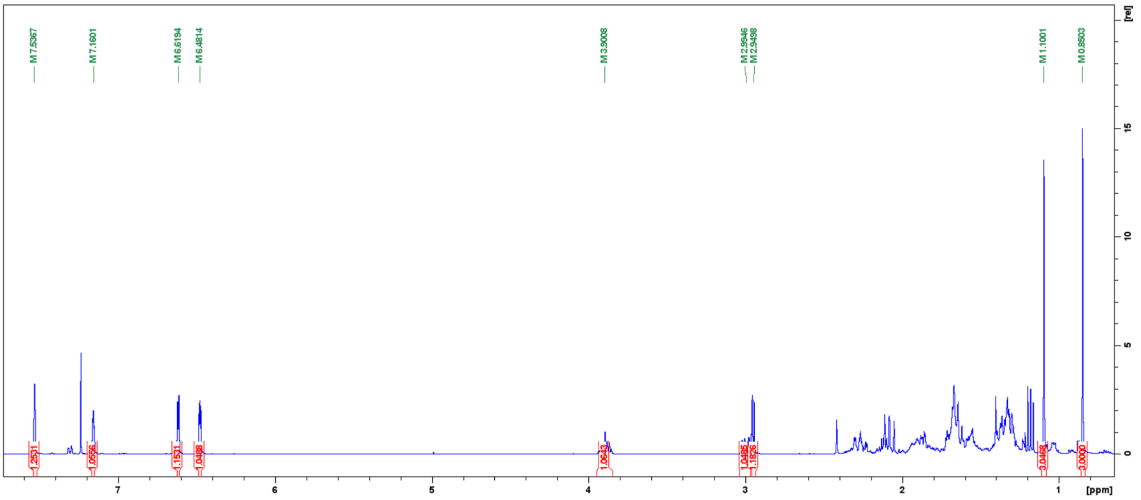

^{13}C NMR spectrum of compound 20

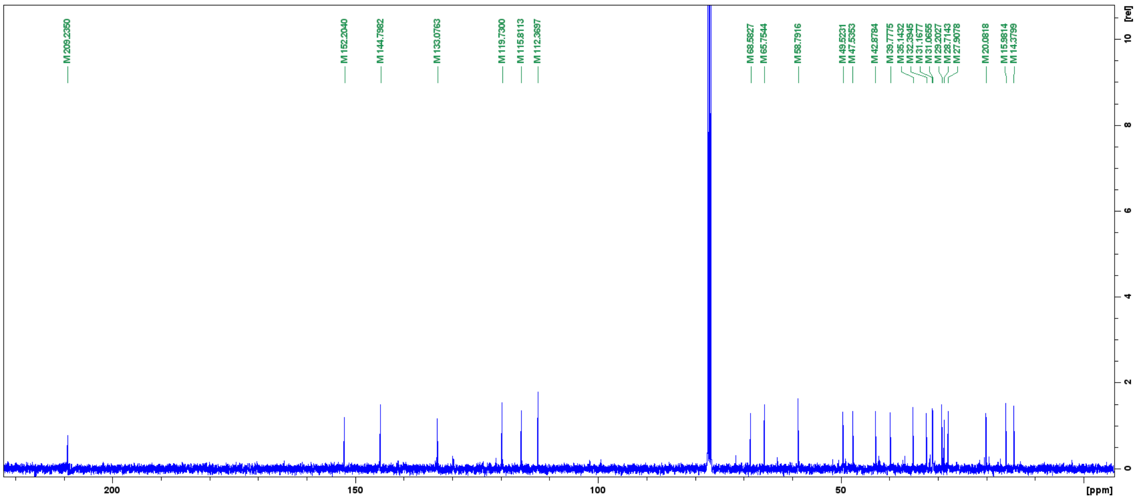

^{1}H NMR spectrum of compound 21

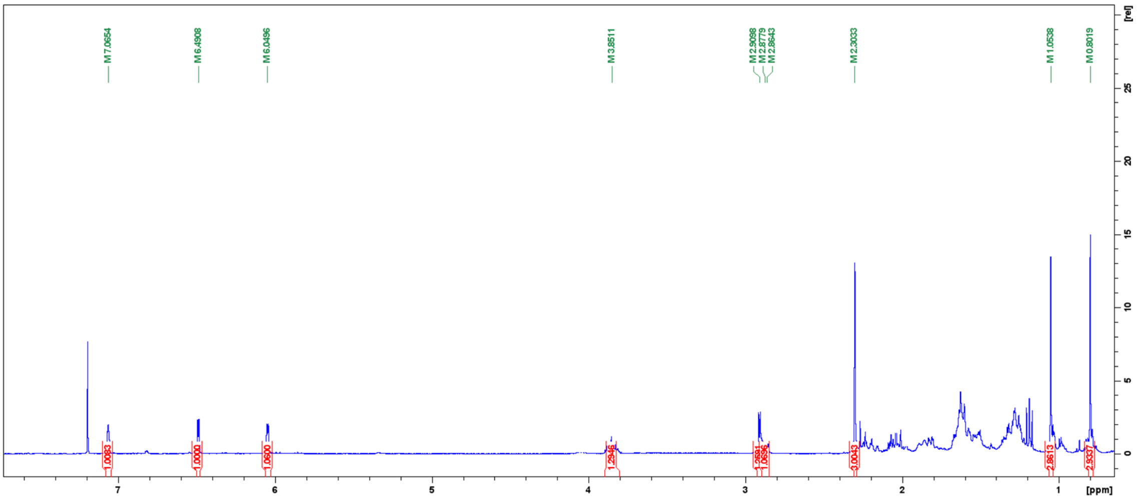

^{13}C NMR spectrum of compound 21

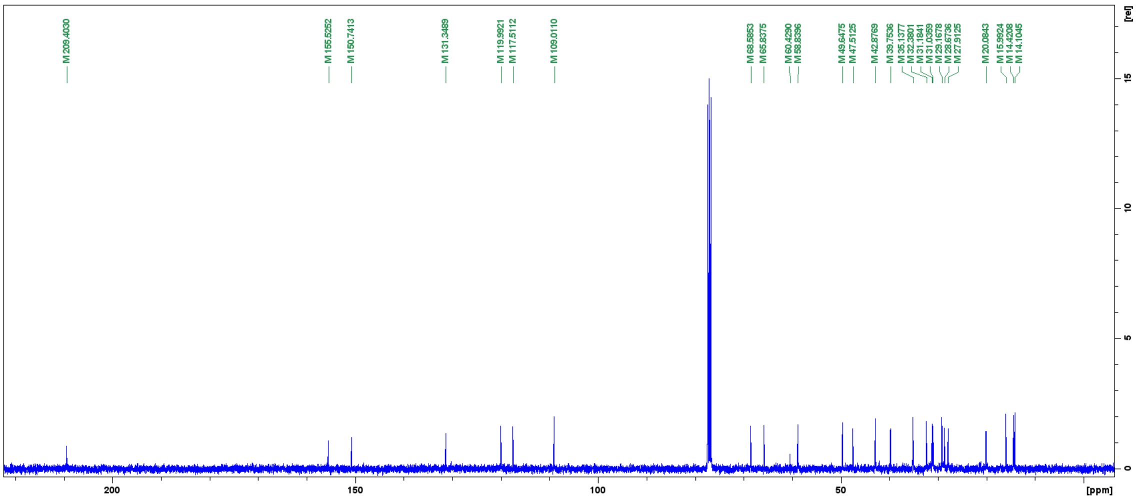

^{1}H NMR spectrum of compound 22

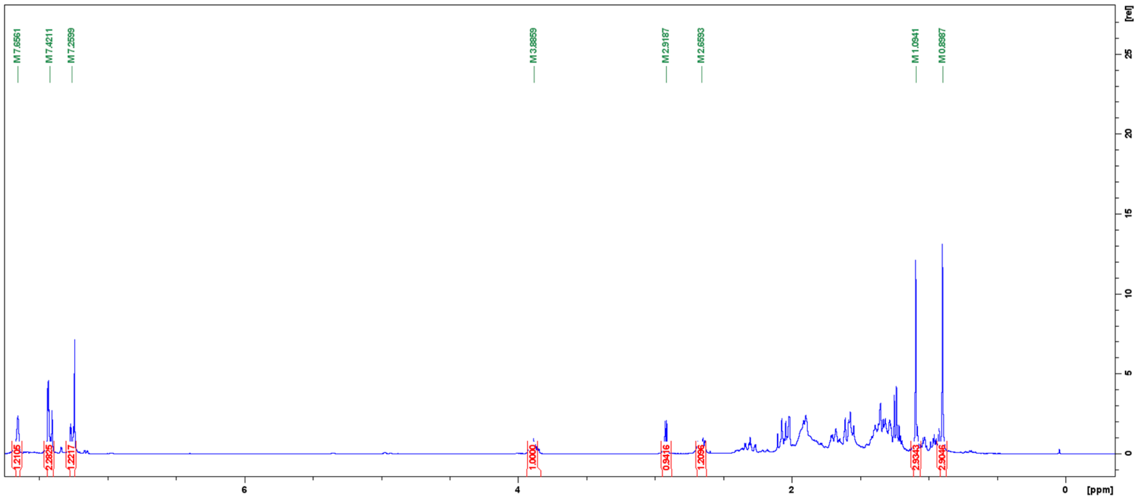

^{13}C NMR spectrum of compound 22

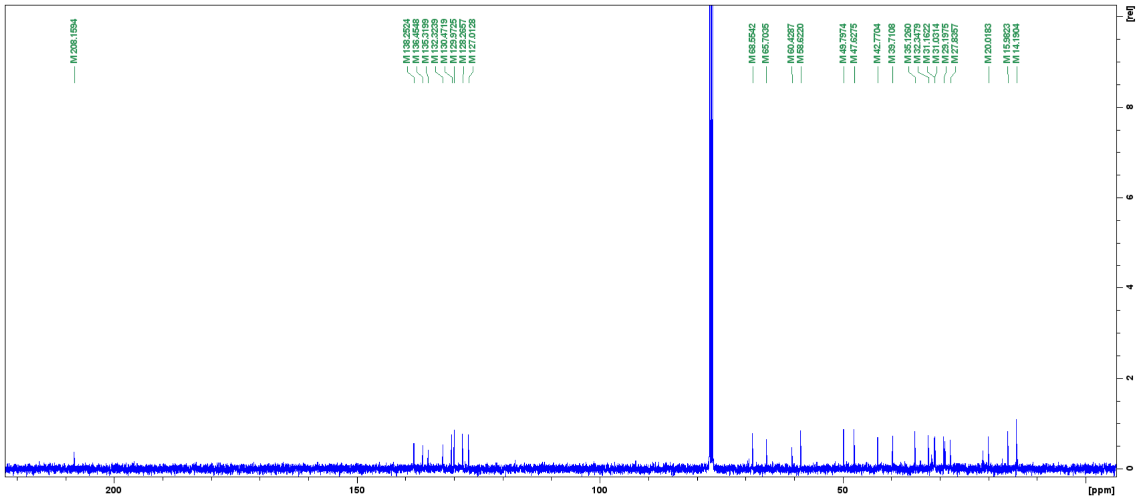

^{1}H NMR spectrum of compound 23

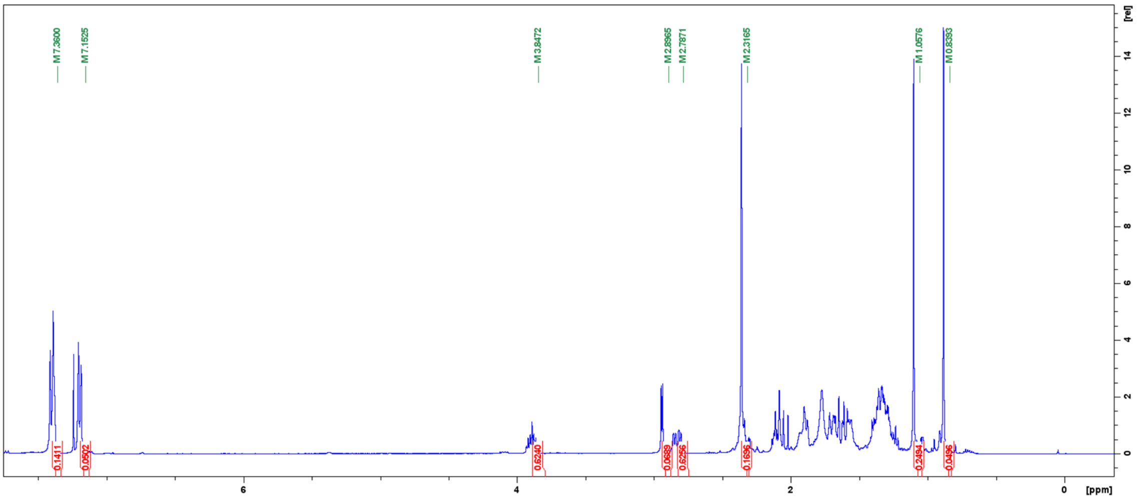

^{13}C NMR spectrum of compound 23

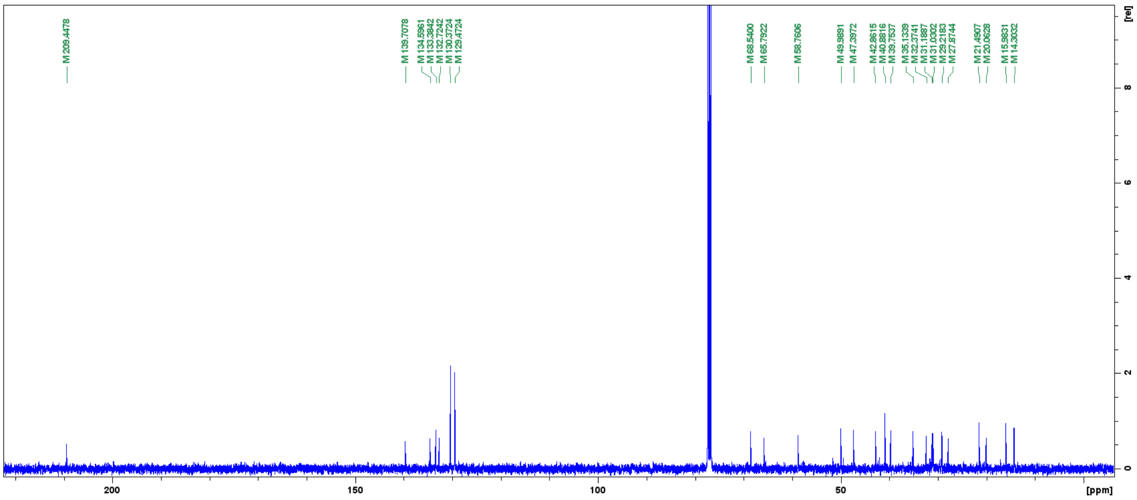

¹H NMR spectrum of compound 24

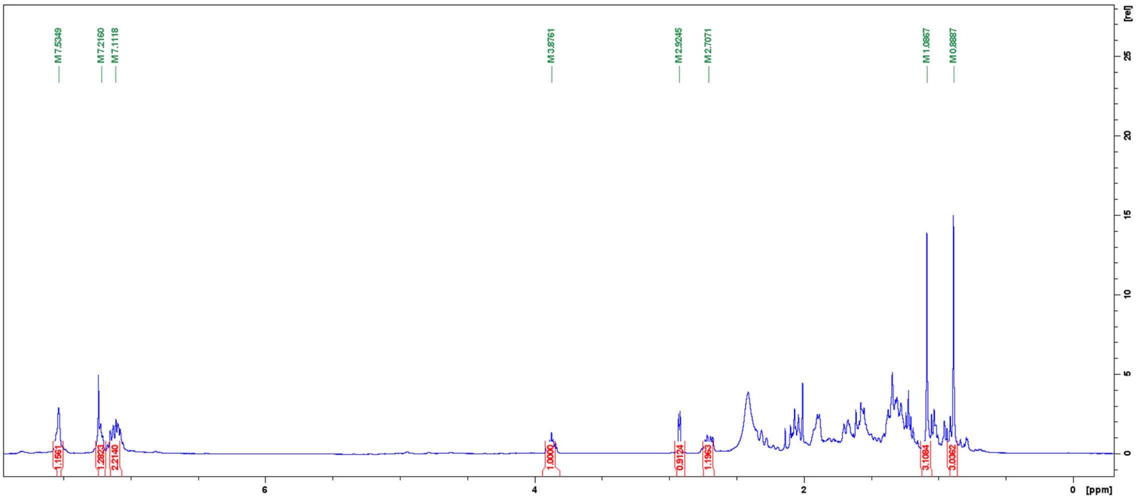

¹³C NMR spectrum of compound 24

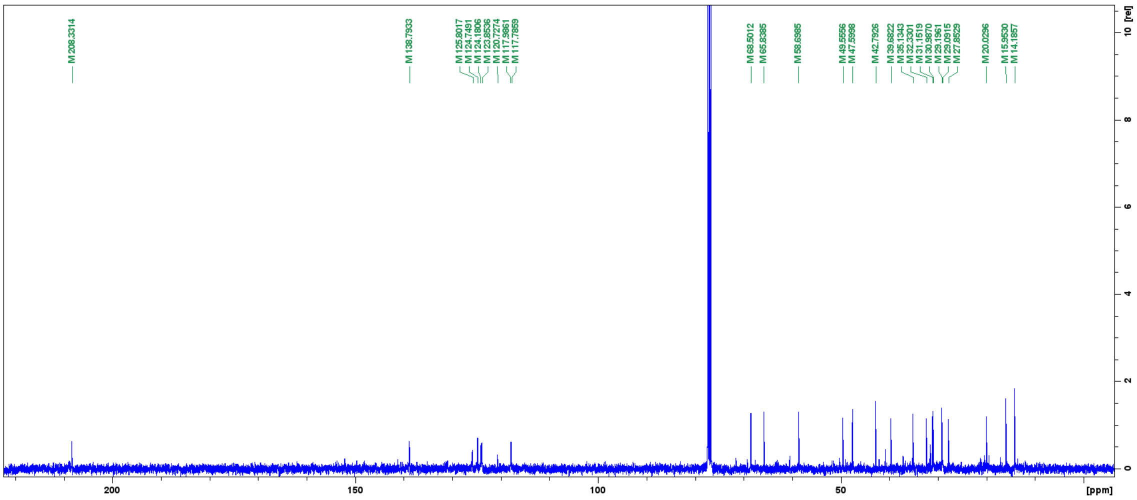

^{1}H NMR spectrum of compound 25

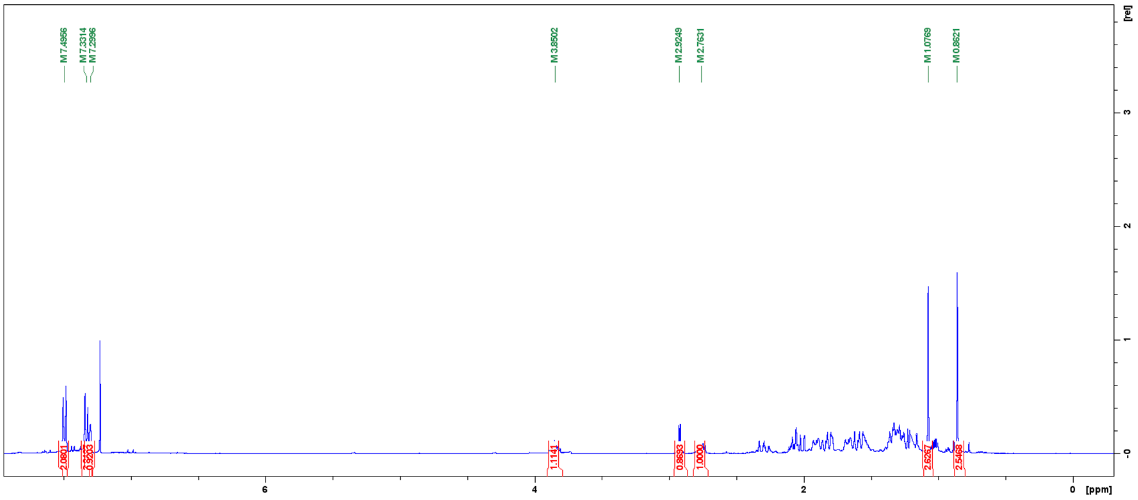

^{13}C NMR spectrum of compound 25

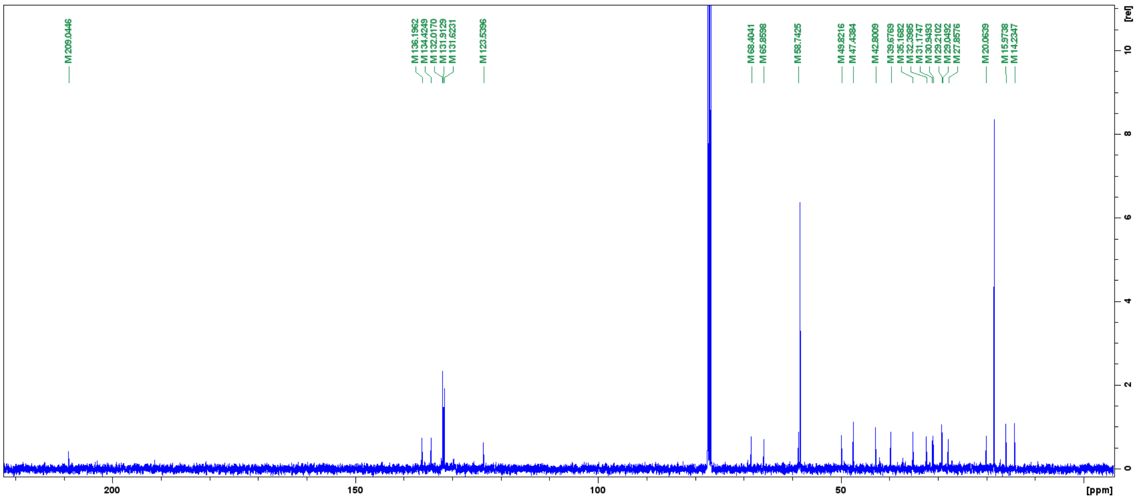

^{1}H NMR spectrum of compound 26

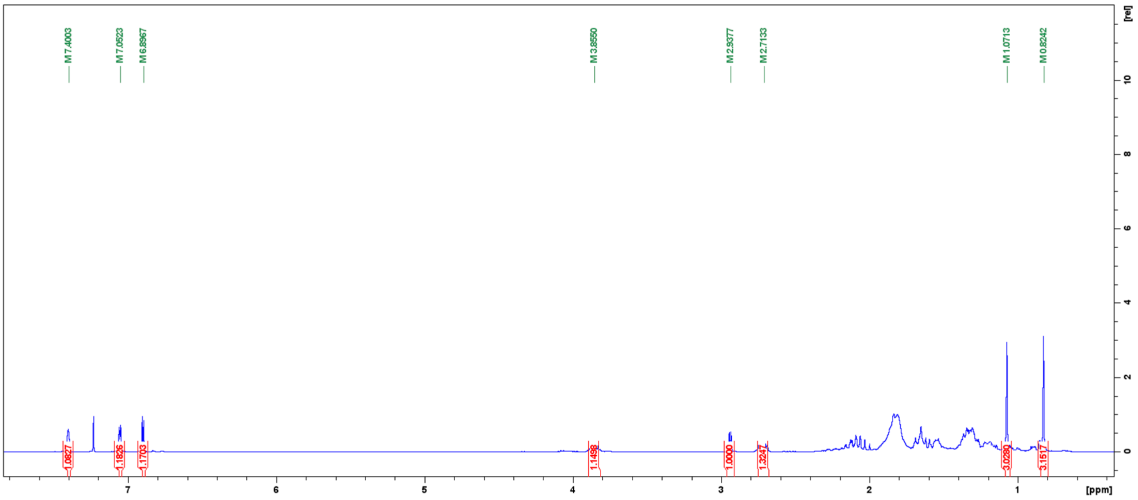

^{13}C NMR spectrum of compound 26

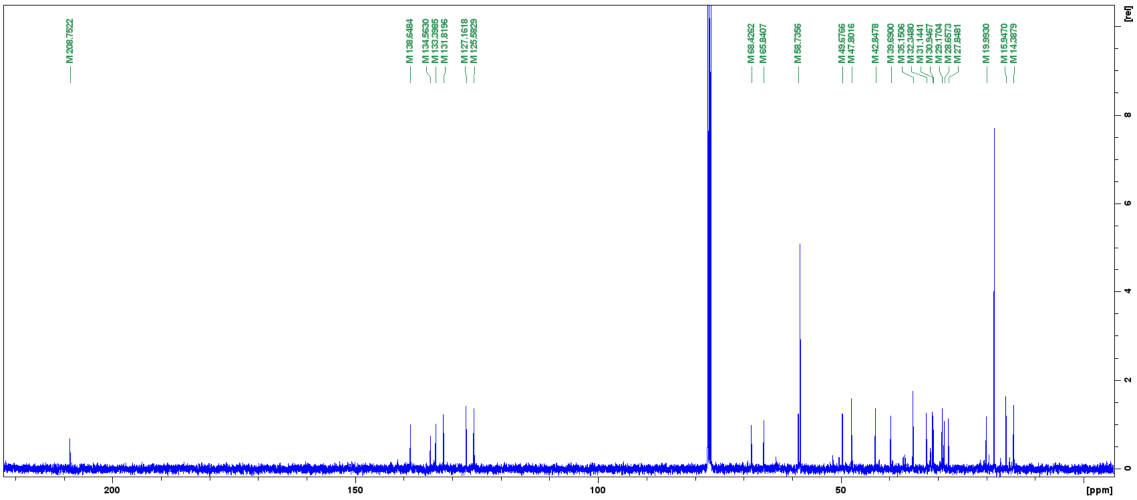

¹H NMR spectrum of compound 27

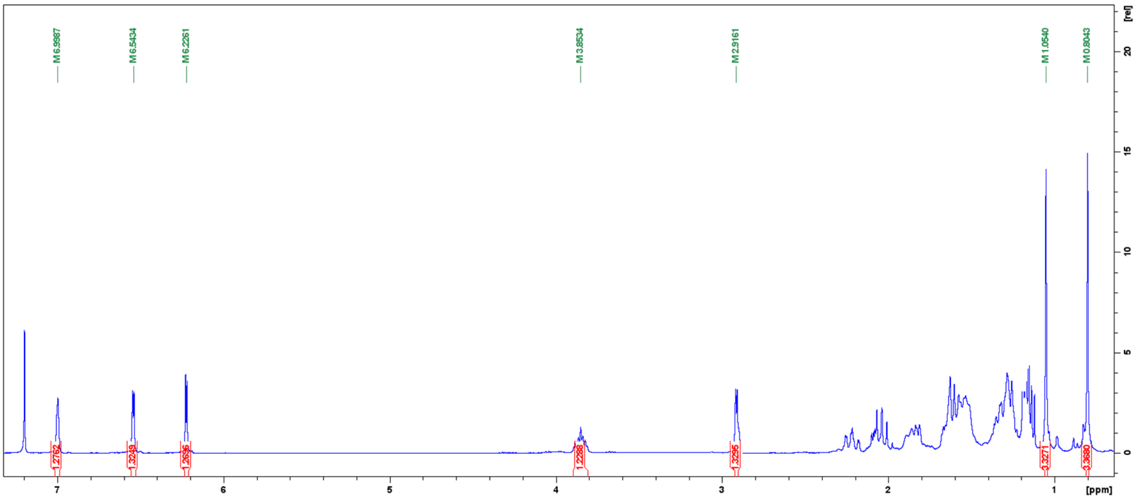

¹³C NMR spectrum of compound 27

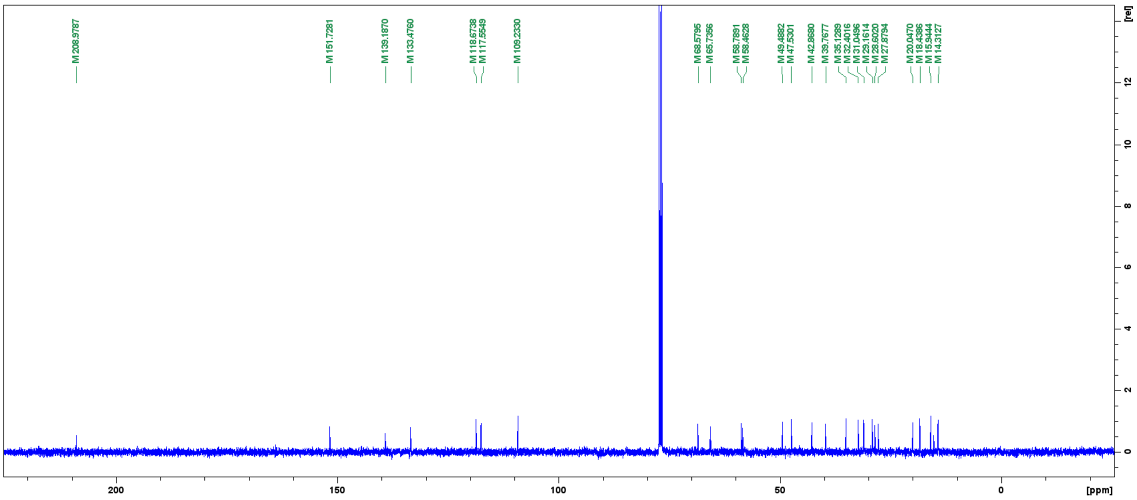

S2. Additional biological data

The most representative photograph shots of MCF-7 cells after 48 h of treatment with 5-FU and steroid **19** at 1 and 10 μM, and respective negative control (optical microscope coupled with a digital camera, zoom: 100 ×).

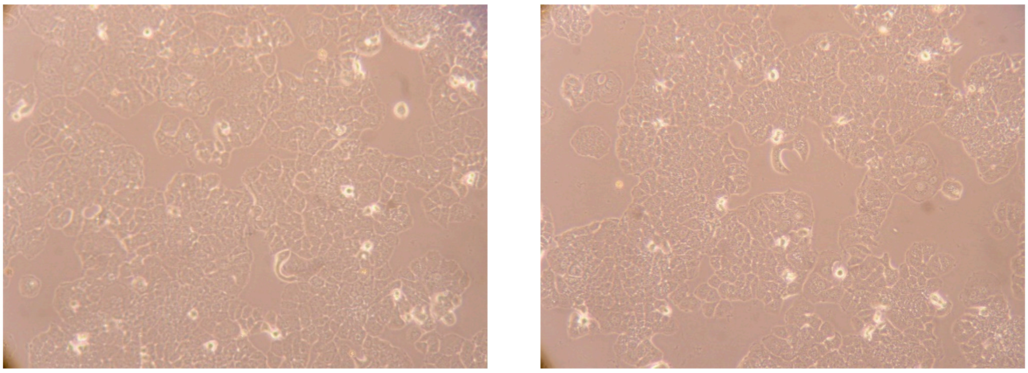

Negative control, 48 h

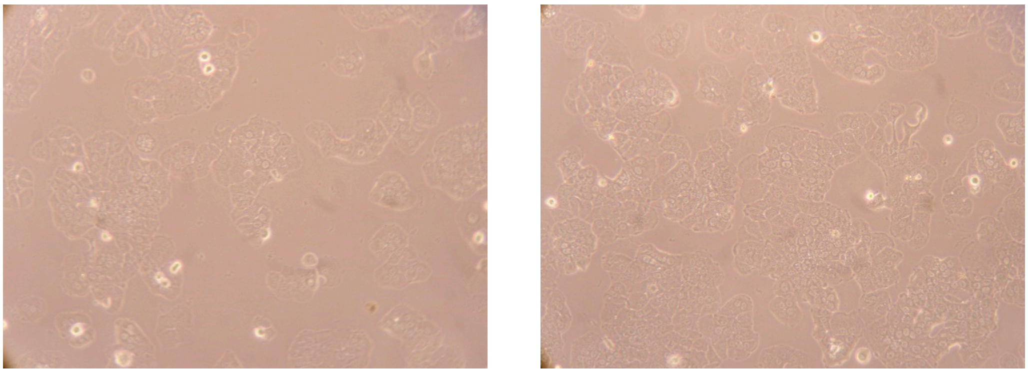

5-FU, 1 μM, 48 h

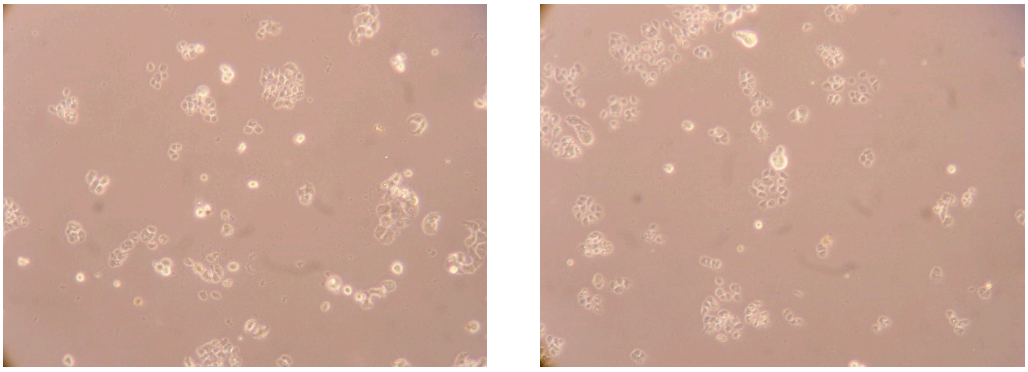

5-FU, 10 μM, 48 h

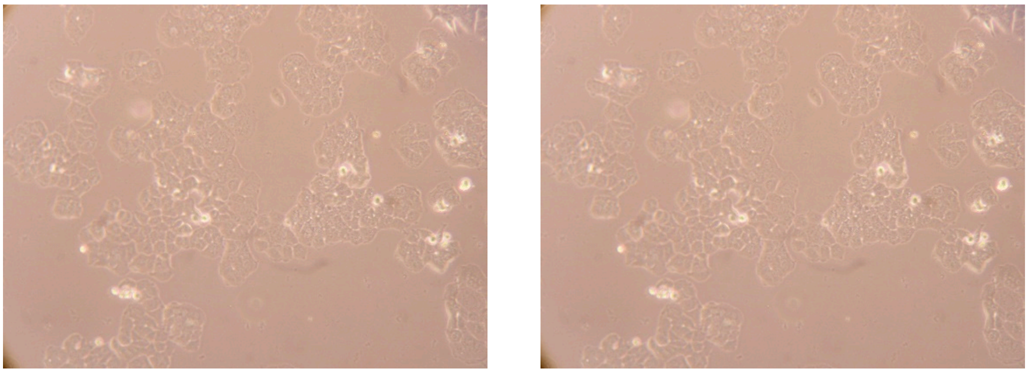

19, 1 μM, 48 h

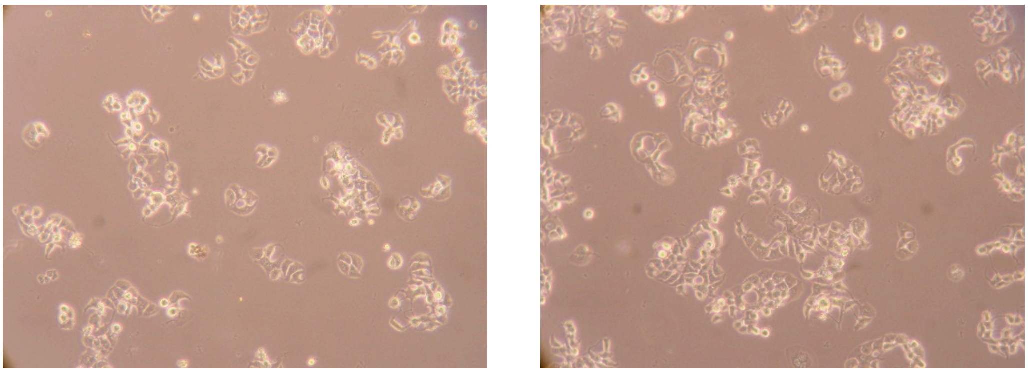

19, 10 μM, 48 h

S3. Concentration-response curves for steroid 19 and IC_{50} determination additional data

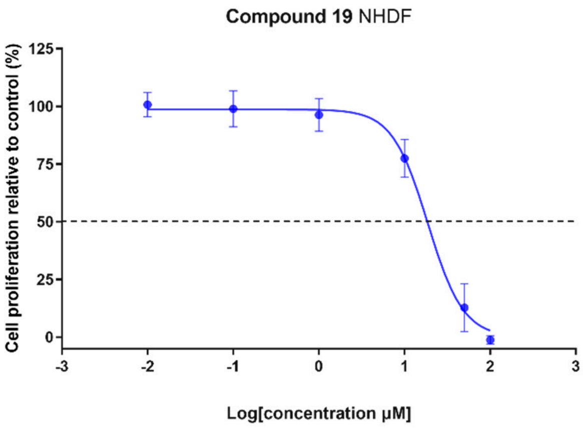

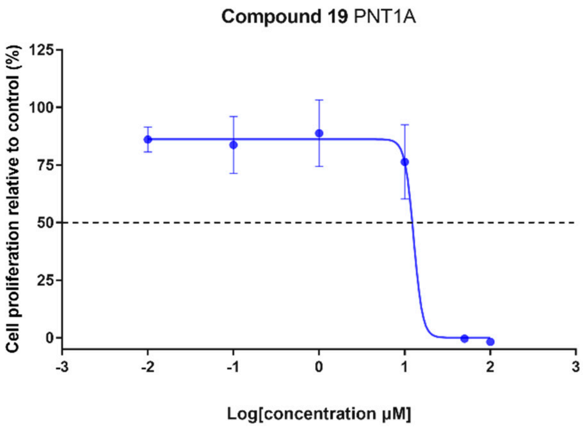

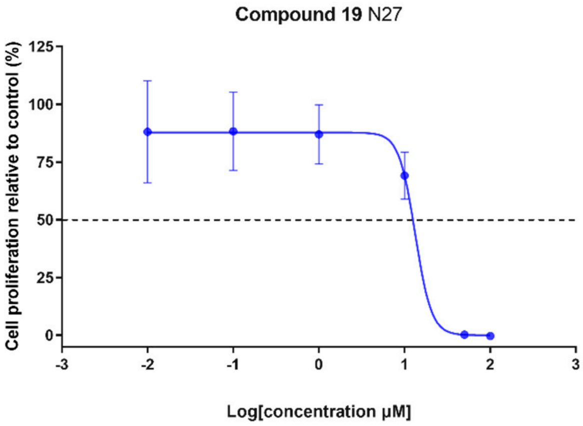

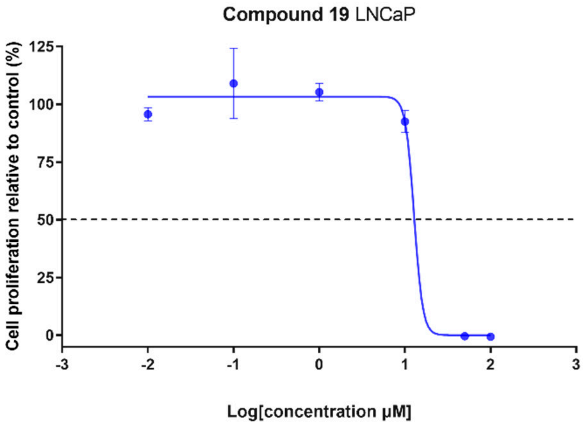

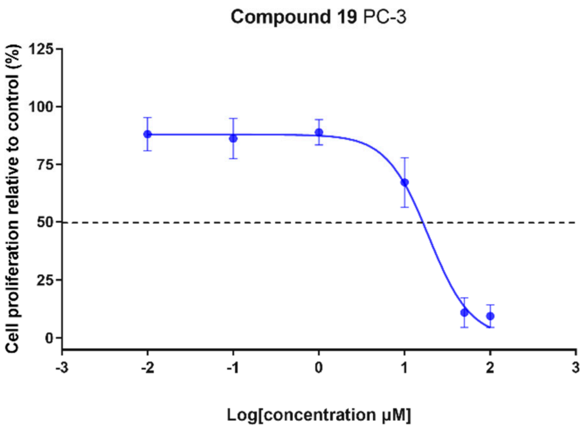

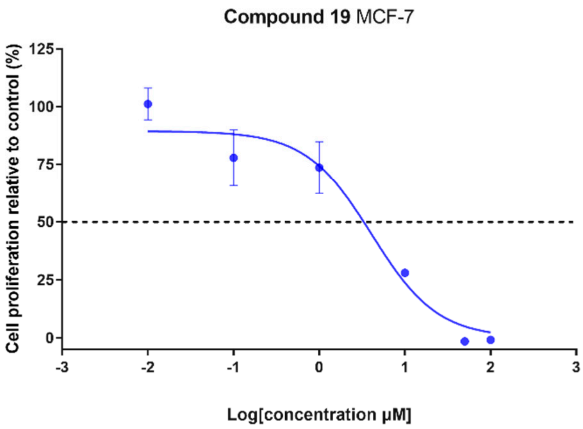

The following table shows the estimated IC_{50} values (μM) and respective R^2 for final products and 5-FU in non-tumoral cells (NHDF, PNT1A, and N27), and in tumoral cells (LNCaP, PC-3, and MCF-7).

Compound	NHDF		PNT1A		N27		LNCaP		PC-3		MCF-7	
	IC_{50}	R^2	IC_{50}	R^2	IC_{50}	R^2	IC_{50}	R^2	IC_{50}	R^2	IC_{50}	R^2
**5-FU**	6.34	0.95	6.48	0.95	3.19	0.87	9.43	0.95	21.20	0.97	6.30	0.90
**15**	39.52	0.94	33.12	0.96	9.21	0.91	27.37	0.95	17.02	0.99	42.67	0.84
**16**	39.27	0.96	54.52	0.94	33.18	0.91	-	-	20.06	0.99	-	-
**17**	32.33	0.92	>100	-	>100	-	-	-	-	-	20.82	0.83
**18**	29.65	0.94	29.56	0.84	12.58	0.86	-	-	37.56	0.98	-	-
**19**	18.69	0.97	12.75	0.94	13.32	0.91	12.83	0.98	19.03	0.96	3.47	0.93
**20**	49.96	0.92	56.63	0.92	64.46	0.91	-	-	-	-	-	-
**21**	23.44	0.94	46.33	0.93	13.16	0.85	-	-	-	-	-	-
**22**	14.95	0.96	14.41	0.99	16.77	0.86	15.02	0.98	44.46	0.99	14.46	0.90
**23**	28.34	0.96	13.27	0.98	12.74	0.89	19.29	0.98	13.97	0.99	18.68	0.93
**24**	33.43	0.86	56.46	0.95	18.92	0.92	-	-	-	-	-	-
**25**	15.98	0.93	15.33	0.98	15.36	0.90	36.88	0.88	14.52	0.98	14.52	0.92
**26**	16.62	0.85	15.58	0.99	14.34	0.96	11.34	0.97	43.76	0.97	21.82	0.92
**27**	18.40	0.83	19.47	0.95	42.35	0.90	15.80	0.93	-	-	56.90	0.92

S4. Interactome of steroid 22 at aromatase active site

Interactions of steroid **22** at aromatase active site:

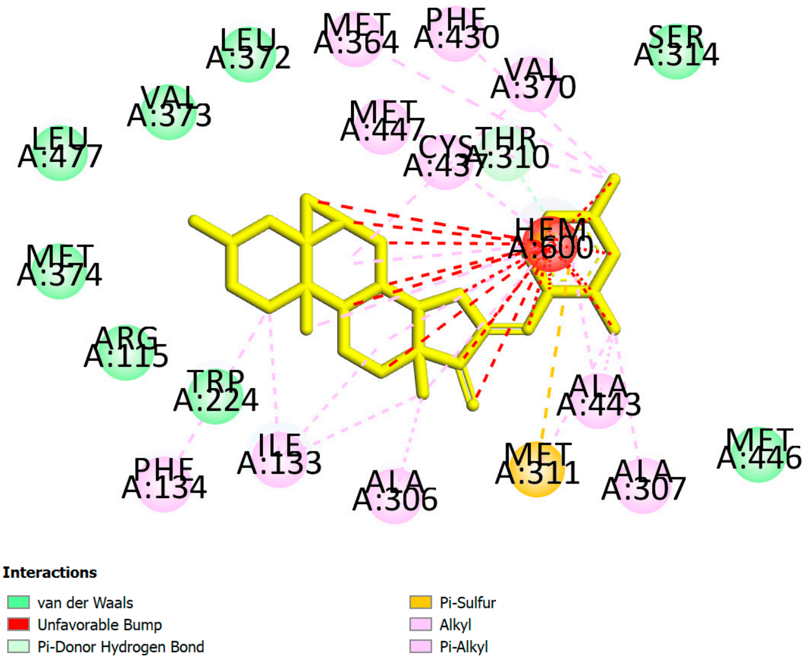

Interactions of androstenedione (co-crystallized ligand) at aromatase active site:

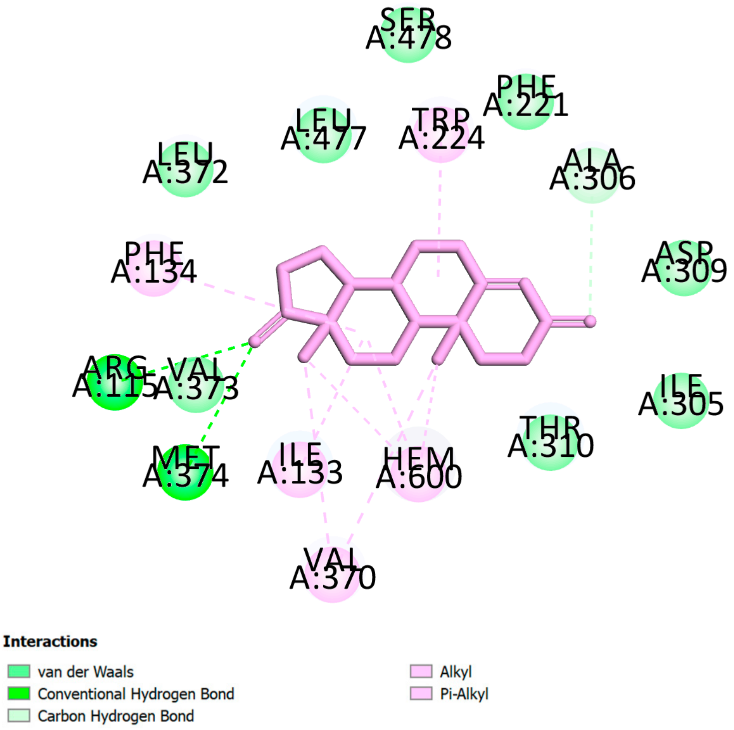

Overlap of **22** (yellow) and androstenedione (purple) in the active site of aromatase:

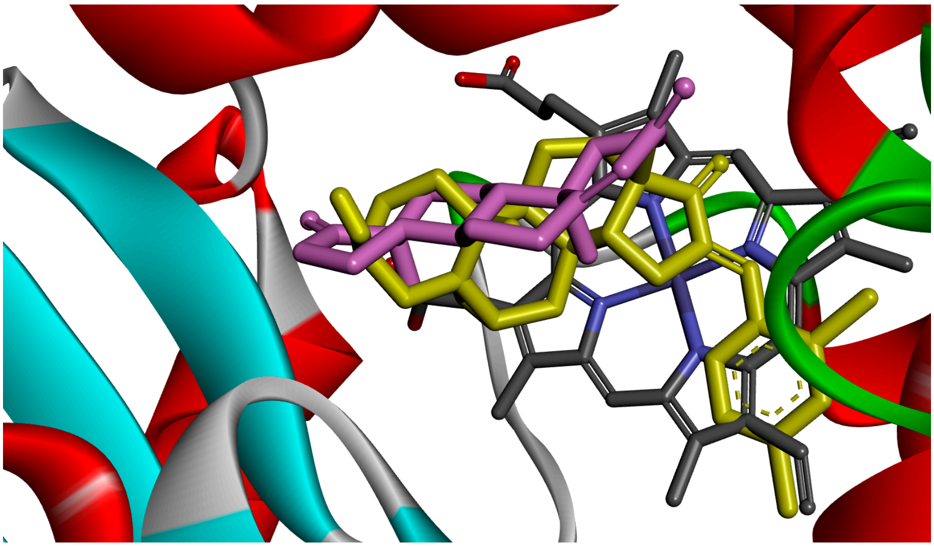

Supplement: Supplementary file 1 [file biomedicines-11-00812-s001.zip › biomedicines-2247188-supplementary.pdf]
